# The Structure and Phenology of Non-Native Scolytine Beetle Communities in Coffee Plantations on Kauaʻi

**DOI:** 10.3390/insects9040123

**Published:** 2018-09-20

**Authors:** Jared Bernard, Curtis P. Ewing, Russell H. Messing

**Affiliations:** 1 Kauaʻi Agricultural Research Center, University of Hawaiʻi–Mānoa, 7370 Kuamoʻo Road, Kapaʻa, HI 96746, USA; messing@hawaii.edu; 2 Research Extension Center, University of Hawaiʻi–Mānoa, 875 Komohana Street, Hilo, HI 96720, USA; curtisewing1@gmail.com

**Keywords:** community ecology, diversity, spatiotemporal distribution, phenology, Scolytinae, invasive species

## Abstract

Populations and communities are known to respond to abiotic conditions, but the forces determining the distribution of particular insect pests are sometimes overlooked in the process of developing control methods. Bark and ambrosia beetles (Curculionidae: Scolytinae) are important pests of crops, forestry, and ecosystems worldwide, yet the factors that influence their success are unknown for many species. The Hawaiian archipelago is host to over three dozen invasive scolytines, many of which occur on Kauaʻi and are pests of agriculture. We analyzed scolytine community dynamics at two coffee estates: a hand-harvested site in a tropical wet forest and a mechanically harvested site in a tropical dry savanna. Our regression analyses show overall scolytine abundance was negatively correlated with rainfall, as were four species: the tropical nut borer (*Hypothenemus obscurus*), *H. brunneus*, *Cryphalus longipilus*, and *Xyleborinus andrewesi*. These relationships contributed to the compositions of the communities being markedly dissimilar despite having the same species richness. Multivariate analysis found no influence from temperature or harvest method on community dynamics. This information can be valuable for the timing of pest control methods, for predicting the success of possible new scolytine arrivals on Kauaʻi, and for forecasting how these species may spread with climate change.

## 1. Introduction

Abiotic factors influence seasonal variation of populations and thus the structure of their communities (e.g., diversity). Specific knowledge of these interactions is invaluable to understanding species geographical distributions and fluctuations of abundances for the purposes of ecological modeling, developing conservation strategies for threatened species, and directing management of pest species [1,2,3,4]. Many ambrosia and bark beetles (Curculionidae: Scolytinae) are important global pests of natural ecosystems, forestry, and agriculture, requiring tremendous financial and human resources to combat them [5,6,7,8,9]. Prominent examples include the mountain pine beetle, *Dendroctonus ponderosae* Hopkins, which has devastated millions of hectares of ecologically and commercially important pines in the Pacific Northwest and Rocky Mountains [10]; the black twig borer, *Xylosandrus compactus* (Eichhoff), which is a pest of hundreds of crops, ornamental plants, and forest trees [11]; and the coffee berry borer, *Hypothenemus hampei* (Ferrari), which is the most impactful pantropical pest of coffee production [12]. Much attention has been devoted to elucidating life histories of scolytines in the context of damage to host plants, and to developing monitoring and control techniques (i.e., chemical and biological) [5,11]. Yet understanding how abiotic factors (e.g., rainfall, temperature) determine their geographical or seasonal success can aid in targeting management resources [2,4,13]. This information can also lead to predictive distribution modeling to forecast the establishment of incipient pests from globally traded crops or wood products [3,7] or to account for climate change [12].

The Hawaiian Archipelago has ~58 species of scolytines, which includes 21 endemic species of *Xyleborus* Eichhoff (~10 of which are native to more than one island) [14,15]. It has been invaded by numerous widespread pest species, including five *Xyleborus* species, five species of *Coccotrypes* Eichhoff, the tea shot-hole borer (*Euwallacea fornicatus* [Eichhoff]) and one other congener, *Xylosandrus compactus* plus three other congeners, and ~12 species of *Hypothenemus* Westwood. To add to that, *H. hampei* was detected invading coffee in the Kona District on Hawaiʻi Island in September 2010 [16]. It was subsequently recorded in the Kaʻū District of Hawaiʻi Island in May 2011, Dole’s Waialua Estate on Oʻahu in December 2014, and on Maui in December 2016. The richness of the non-native scolytine community in the Hawaiian Islands is thus ideal for analyzing responses of different species to various environmental variables.

The largest coffee estate in the United States is the Kauaʻi Coffee Company with 1254.5 ha on the Hawaiian island of Kauaʻi, where *H. hampei* has not yet been discovered. Nonetheless, *X. compactus* is also known to be a pest of coffee [11] and is established on Kauaʻi, as are many other non-native scolytines. Authors have previously addressed the effect of host availability on scolytine communities [6,8,17]; monocultures of crops present an opportunity to factor out aspects of the community structure related to hosts and focus on the abiotic factors. Uncovering the drivers of phenology and structure of invasive scolytine communities on Kauaʻi can also inform management strategies and indicate the potential success of *H. hampei* or other adventive scolytines, and therefore would be highly useful to protecting Kauaʻi’s crops.

Like the other Hawaiian Islands, the movement of trade winds across Kauaʻi’s volcanic topography creates a range of climate patterns [18], yielding variable growing conditions across the island. The Kauaʻi Coffee Company is located on the drier south side, but in other climatic conditions are other small coffee farms, such as Moloaʻa Bay Coffee on the wetter windward side, as well as several naturalized coffee stands. Because Kauaʻi is a relatively small island, the immediate access to variable climate regimes makes Kauaʻi a natural laboratory for testing community responses. Furthermore, the Kauaʻi Coffee Company is mechanically harvested, whereas Moloaʻa Bay Coffee is hand-harvested, permitting us to additionally question whether different harvesting methods influence the bark beetle community. By collecting samples for two growing seasons at the Kauaʻi Coffee Company and one season at Moloaʻa Bay Coffee, this study compared the species composition of scolytine communities at both sites. To understand how abiotic forces influence scolytines at these sites, we analyzed the fluctuating abundances over time for each species and the community as a whole for correlations with rainfall, temperature, and harvest techniques. We furthermore assessed the correlations between scolytine species to explore interspecies determinants of community structure.

## 2. Materials and Methods

### 2.1. Study Sites

The Kauaʻi Coffee Company is located in the hamlet of Numila on the southern coast of Kauaʻi between Hanapepe River and the Kalaheo Gulch (UTM: 4Q 442039 E, 2421806 N). The visitor center is situated at the center of the estate along Highway 540 at ~87.2 m elevation. Because the weather stations around Numila have incomplete data for both the sampling period and the 1981–2010 climate normals, the station from the adjacent district of Kōloa (GHCND: US1HIKI0020) was used. According to the Köppen–Geiger climate classification, the area is a tropical dry savanna (*As*) [19] with an average annual precipitation of 552.7 mm and air temperature of 19.8–28.3 °C, averaging 24.1 °C [20]. The soil at this location is a fine isohyperthermic silty clay loam with a basic igneous parent material ca. 5 million years old [21]. The estate has been used since 1996 for the production of unshaded coffee (*Coffea arabica* L.) cultivars, having previously been used since the mid-nineteenth century for intensive sugarcane (*Saccharum officinarum* L.) cultivation. Arabica varietals raised at Kauaʻi Coffee include typica, blue mountain, and, most predominantly, catuai, a Brazilian hybrid cultivar that has varietals of either yellow or red fruit. The Kauaʻi Coffee Company utilizes mechanical harvesters and processing in an industrial mill, with its annual harvest starting in late September or October and lasting until late December or January, similar to the seasonality in Mesoamerica. This method of harvest strips the berries from the trees, leaving only minute numbers behind. Less accessible terrain on the estate, such as hilltops and gulches, is dominated by haole koa (*Leucaena leucocephala* [Lam.] de Wit).

Contrarily, Moloaʻa Bay Coffee uses hand-harvesting more or less year-round. This estate is on the northeast windward shore of Kauaʻi at ~80 m elevation in the hamlet of Moloaʻa (UTM: 4Q 466094 E, 2453687 N). The Anahola weather station (GHCND: US1HIKI0021) provided data for the sampling period, while the 1981–2010 climate normals were obtained from the nearby town of Kīlauea. The area receives an average annual precipitation of 1742.9 mm and a temperature of 19.7–26.8 °C, with an average of 23.3 °C [20], giving it a Köppen–Geiger classification of tropical rainforest (*Af*) [19]. Like Numila, this site has very fine isohyperthermic silty clay soil with a basic igneous dust substrate [21]. Moloaʻa Bay Coffee estate is 6.1 ha that includes a mix of shaded and unshaded arabica coffee production, all of which is the typica varietal, interspersed with two rows each of cacao (*Theobroma cacao* L.), Latundan banana (*Musa acuminata* × *balbisiana* silk cultivar), as well as citrus trees. This site also includes hedgerows of vinegartree (*Lophostemon confertus* [R.Br.] Wilson & Waterh.) and elephant-ear tree (*Enterolobium cyclocarpum* [Jacq.] Griseb.). Areas around the estate are dominated by haole koa.

### 2.2. Experimental Design

The sampling period was two growing seasons in Numila: June 2015–March 2016 and June 2016–January 2017. At Moloaʻa, we sampled only during the latter period. We excluded both Junes from statistical analysis, however, because we sampled during only part of the month. We collected scolytines in Brocap^®^ traps (Cirad, Montpellier, France). To maintain the efficacy of the traps, every three months we replaced each trap’s 3:1 methanol–ethanol lure (Scentry Biologicals, Inc., Billings, MT, USA) and 2.5 × 2.5 cm Vaportape^TM^ II insecticidal strip (2,2-dichlorovinyldimethylphosphate; Aberdeen Road Company, Emigsville, PA, USA). Despite the recommendation by Scentry Biologicals, Inc. to replace the lures every three weeks, we found them to be effective for at least three months, with no impact on collection (data not shown). Although intended for surveying for *H. hampei* [22], this trapping system proved to be a non-specific means for collecting not only nearly two dozen species of scolytines, but also many other pests of agriculture, including several species each of nitidulids, bostrichids, anthribids, cerambycids, and coccinellids, as well as many hemipterans, hymenopterans (including sphecids, ants, and numerous parasitoids), blattellids, dipterans, lepidopterans, and other insects (data not shown). We hence used only these traps, thereby standardizing our collection methods to avoid accounting for variable trapping methods in our data. We placed the traps to encapsulate as much of each property as possible. At the Kauaʻi Coffee Company at Numila, we placed 12 traps: seven in the fields at the center of the property, two flanking the hopper at the mill (to encounter specimens transported from distant parts of the property), and three at the corners of the estate. We positioned two traps at opposite ends of the Moloaʻa Bay Coffee property, which is proportionally adequate given that this estate is 0.5% the size of the estate at Numila. To ameliorate potential edge effects, traps near the perimeters of each estate were a minimum of 6 m from the limits of coffee production, beyond which was another 3–10 m of buffer to the edge of the property. The predominant haole koa surrounding each estate would further help to mitigate differences between sites.

### 2.3. Analysis

After identifying specimens following Wood [23,24], Beaver and Maddison [25], and Samuelson [14], we preserved them in 95% EtOH and verified vouchers prior to accessioning at the University of Hawaiʻi Insect Museum (UHIM) in Honolulu (listed in S1). Because the area and number of traps differed between the sites, we calculated the similarity between the communities using relative abundances with the Bray–Curtis dissimilarity index. The Shannon–Wiener index enabled us to assess the diversity because it can be related to species richness to determine the similarity of species’ abundances or species evenness. As this provides a snapshot of the community structure, we plotted these metrics over time to evaluate the communities’ structure in the context of phenology. We assessed log-transformed data (*x’* = log_10_[*x* + 1]) for normality using the Shapiro–Wilk test and for skewness using Pearson’s coefficient before conducting regression analyses with R 3.5.0 [26] to discern correlation of the communities’ phenology to abiotic factors, both within and among sites. When both beetle and environmental data were normally distributed, we used parametric linear regression, and for the others we used Kendall’s *τ_b_* correlation. Multiple analysis of variance (MANOVA) tests enabled us to further assess any potential interactive effects of the variables. Finally, to analyze additional influences of the structure of the communities from beetle–beetle interactions, we constructed a scale-free correlation network using the R package “igraph” [27] with log-transformed abundance data. With the aid of the R package “Matrix” [28], we filtered the correlation network for significant *p*-values. We projected the networks using the Fruchterman–Reingold algorithm because it employs natural repellent forces to display distances between nodes [29].

## 3. Results

**Figure 1:**
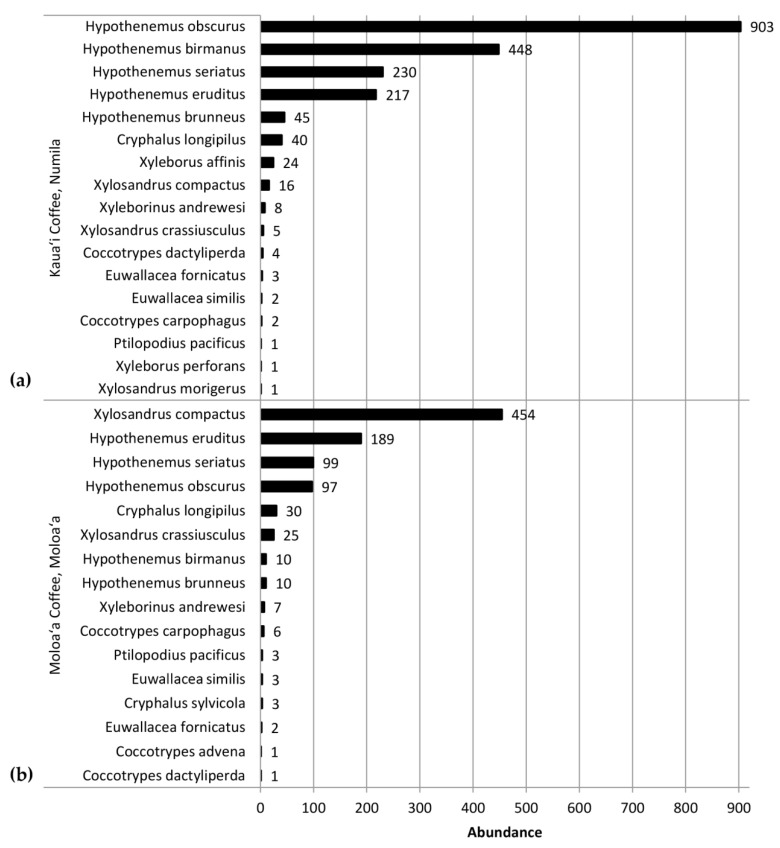
Abundance of scolytines collected at (**a**) the Kauaʻi Coffee Company and (**b**) Moloaʻa Bay Coffee during the 2016–2017 growing season.

**Figure 2:**
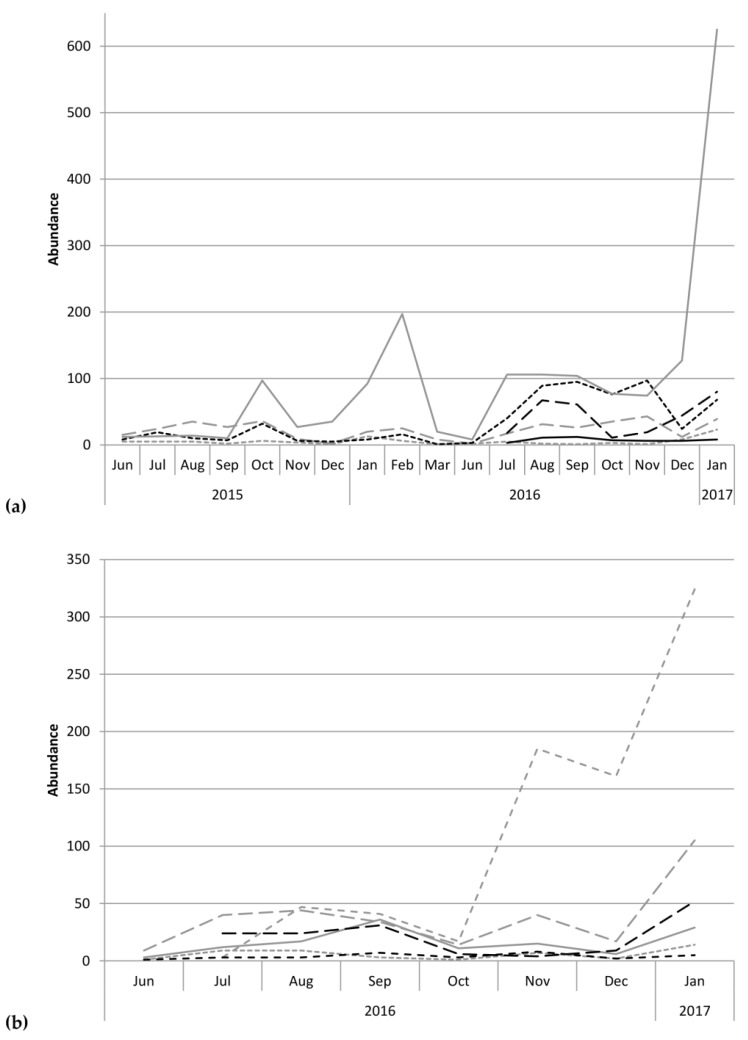
Abundance over time of the six most prominent scolytines at (**a**) the Kauaʻi Coffee Company during the 2015–2016 and 2016–2017 growing seasons, and (**b**) Moloaʻa Bay Coffee during the 2016–2017 growing season. Grey solid = *Hypothenemus obscurus*, black long dashes = *H. seriatus*, black dots = *H. birmanus*, grey medium dashes = *H. eruditus*, grey dots = *Cryphalus longipilus*, black solid = *H. brunneus*, grey short dashes = *Xylosandrus compactus*, black short dashes = *X. crassiusculus*.

**Figure 3:**
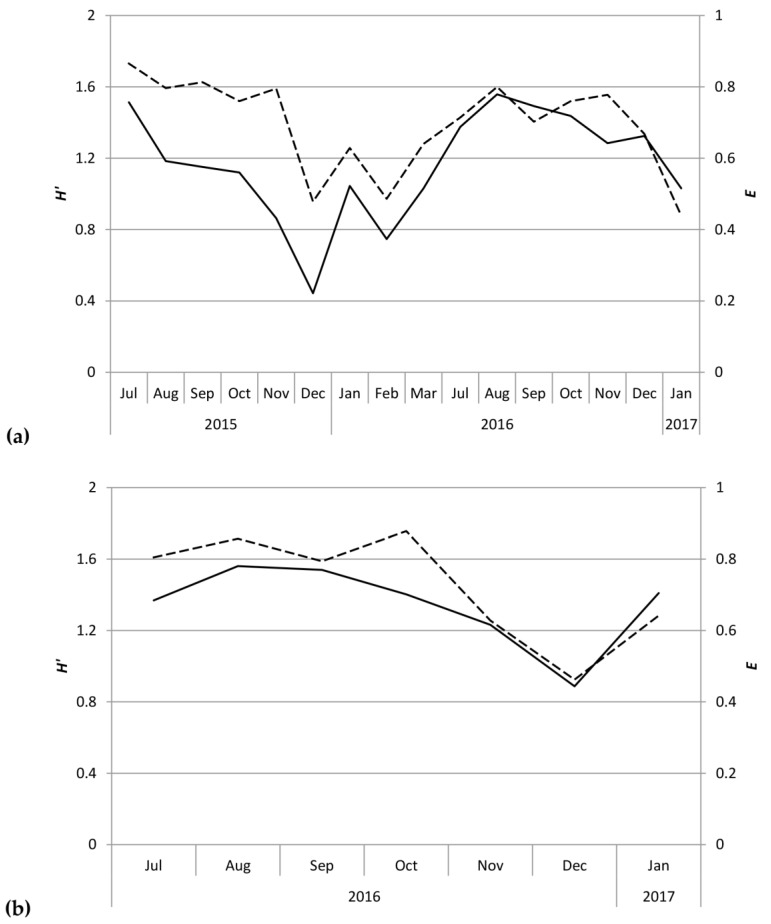
Shannon–Wiener diversity (*H’* = solid line) and species evenness (*E* = dashed line) of scolytine community at (**a**) the Kauaʻi Coffee Company and (**b**) Moloaʻa Bay Coffee.

This study identified 20 species of scolytines, with 4841 specimens collected and preserved (raw data deposited on Figshare.com). Three of these species are new records for Kauaʻi and are listed in Supplemental 1. Although both Moloaʻa Bay Coffee and the Kauaʻi Coffee Company had an overall species richness of 17, species composition differed between the sites. Using relative abundance to account for different trap numbers, the Bray–Curtis dissimilarity index was 72.89, so the communities were 27.11% similar. At Moloaʻa, the dominant species was *Xylosandrus compactus* in tribe Xyleborini, whereas the dominant species in Numila was the tropical nut borer, *Hypothenemus obscurus* (Fabricius) in tribe Cryphalini (Figure 1 and Figure 2). We calculated the Shannon–Wiener diversity index (*H’*) using monthly totals to describe changes to the community structure over time, and superimposed over this is the monthly species evenness (*E*), based on species richness, for both Moloaʻa and Numila (Figure 3).

Relative humidity data were lacking during the sampling period at both sites, and so were excluded from the analysis aside from the inference via rainfall (rain being 100% RH). Therefore, the abiotic factors analyzed in this study included temperature, precipitation, and harvest techniques. Temperature was not significantly different between the two sites (*F*_(1,11.09)_ = 3.98, *p* = 0.07); as temperature remained relatively stable year-round, we found no correlation between precipitation and temperature ((*F*_(1,22)_ = 0.34, *p* = 0.57). Linear regressions also found no statistical trends between temperature and abundance of scolytines, either for the overall communities (*F*_(1,22)_ = 1.85, *p* = 0.19) or for individual species (listed in Table S1). We also note that fluctuation in scolytine abundance did not align with dates on which we replaced the MeOH–EtOH lures (*τ_b_* = −0.12, *p* = 0.50).

**Figure 4:**
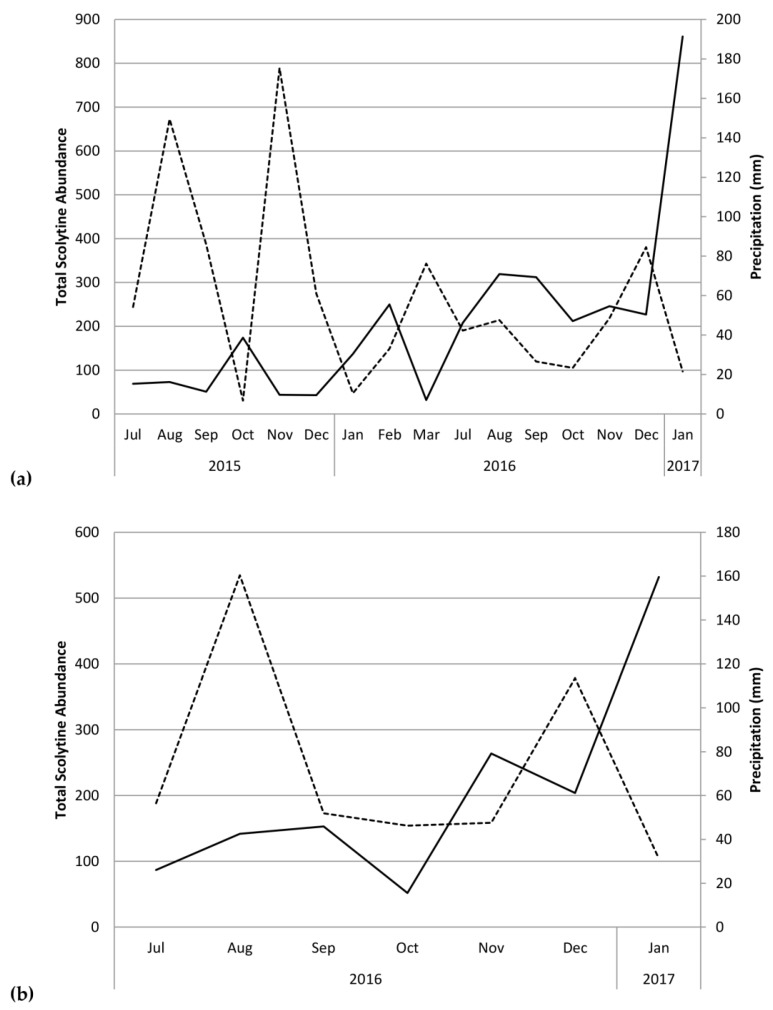
Total monthly scolytine abundance (solid line) and precipitation (dashed line) at (**a**) the Kauaʻi Coffee Company and (**b**) Moloaʻa Bay Coffee.

**Figure 5:**
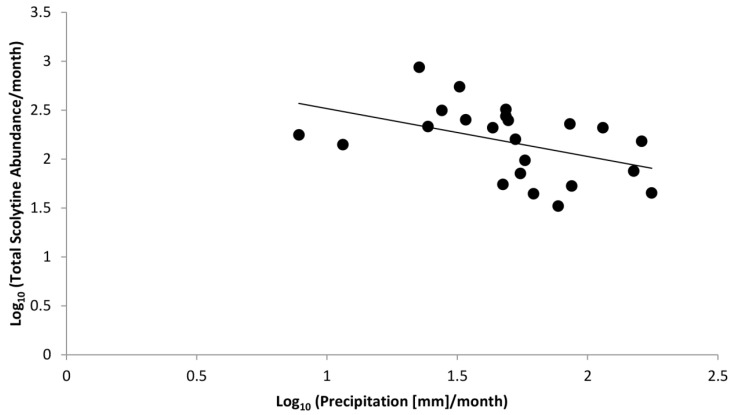
Linear regression of log-transformed data correlating total abundance of scolytines with precipitation.

Figure 4 shows total monthly abundances of scolytines at both Numila and Moloaʻa overlaid against monthly rainfall. Pooled data from Moloaʻa and Numila had normal distribution (*p* = 0.12 for scolytine abundances, *p* = 0.99 for precipitation from nearby weather stations; n = 23 months). Pearson’s coefficient of skewness (*Sk*_2_) also showed that sample distribution was not significantly skewed in either data set (*Sk*_2_ = −0.27 for scolytines, *Sk*_2_ = 0.38 for rainfall), both being less than twice the standard error of the skewness, and their variances were equal (*F* = 1.20, critical value = 2.04). A linear regression revealed a negative correlation between total scolytine abundance and precipitation (*F*_(1,22)_ = 12.21, *p* = 0.00) with Pearson’s correlation coefficient *r* = −0.62 and *R*^2^ = 0.38 (Figure 5). When analyzing the sites separately, a significant negative correlation with rainfall also emerged independently at Numila (*F*_(1,15)_ = 4.98, *p* = 0.04, *R*^2^ = 0.28), although the trend did not arise at the wetter Moloaʻa site (*F*_(1,8)_ = 0.00, *p* = 0.97). However, a MANOVA found no significant combined effect of precipitation and temperature on scolytine abundance.

**Figure 6:**
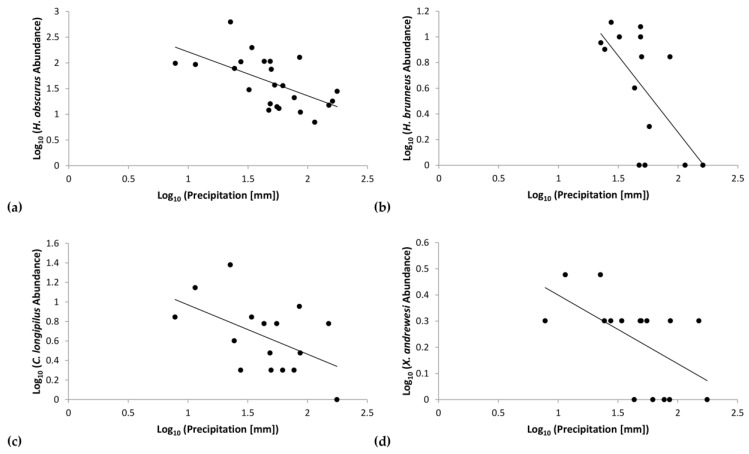
Regression of log-transformed data correlating precipitation and abundance of (**a**) *Hypothenemus obscurus*, (**b**) *H. brunneus*, (**c**) *Cryphalus longipilus*, and (**d**) *Xyleborinus andrewesi*.

Further regression analyses assessed the correlation of individual scolytine species with precipitation. *H. obscurus* had a negative correlation (*F*_(1,21)_ = 10.85, *p* = 0.00) with *r* = −0.58 and *R*^2^ = 0.34 (Figure 6a). Similar trends emerged for the following three species: *H. brunneus* (Hopkins) (*τ_b_* = −0.47, *p* = 0.02; Figure 6b), *Cryphalus longipilus* Schedl (*F*_(1,14)_ = 4.99, *p* = 0.04) with *r* = −0.51 and *R*^2^ = 0.26 (Figure 6c), and *Xyleborinus andrewesi* (Blandford) (*F*_(1,14)_ = 6.65, *p* = 0.02) with *r* = −0.57 and *R*^2^ = 0.32 (Figure 6d). Our analyses did not show significant trends with rainfall for the other species.

To compare seasonal fluctuations in the community at Moloaʻa Bay Coffee with the one at Kauaʻi Coffee Company, we assessed only the 2016–2017 growing season when both sites were surveyed (n = 7 months). Relative monthly abundances (i.e., density) were used to eliminate the bias of the sampling area. Although Pearson’s coefficient of skewness indicated that the data were not significantly skewed (*Sk*_2_ = 1.40 for Moloaʻa, *Sk*_2_ = 1.31 for Numila), the Shapiro–Wilk test found the log-transformed data to have a non-normal distribution (*p* = 0.05 for Moloaʻa, *p* = 0.00 for Numila) and unequal variances (*F* = 0.94, critical value = 0.23). The non-parametric Mann–Whitney *U* test rejected significant difference of total monthly scolytine abundances between the two sites (*U* = 23, c.v. = 8). In contrasting monthly species richness (adjusted per trap), we found no difference between Numila and Moloaʻa (*U* = 18, c.v. = 8).

As a test of the relationship of monthly scolytine abundances to harvest at Numila, we calculated the area of remaining coffee fields adjacent to traps from the total area of the Kauaʻi Coffee Company according to their harvest dates. Regression analyses of scolytine abundance against harvested area over two harvest seasons showed no trend, either for the community overall (*F*_(1,9)_ = 0.26, *p* = 0.63) or for individual species. We also found no interactive effects of harvest with temperature and/or precipitation on scolytine abundance (*F*_(1,9)_ = 0.22, *p* = 0.81).

**Figure 7:**
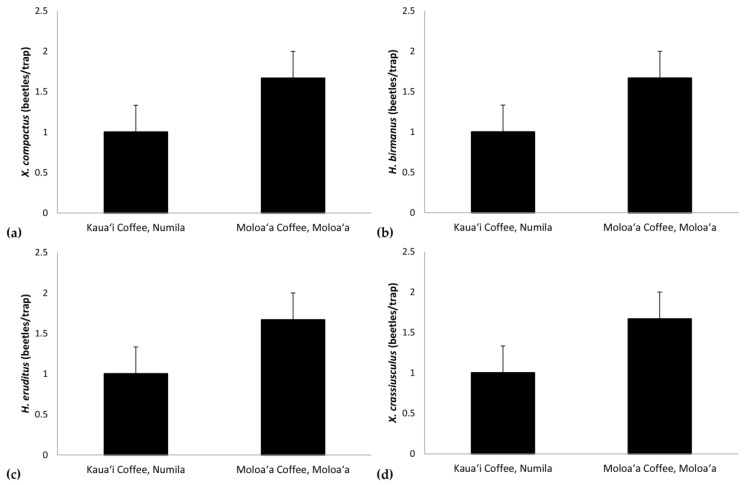
Mean density per trap (± standard error) for (**a**) *Xylosandrus compactus*, (**b**) *Hypothenemus birmanus*, (**c**) *H. eruditus*, and (**d**) *X. crassiusculus* at the Kauaʻi Coffee Company (tropical dry savanna) versus Moloaʻa Bay Coffee (tropical wet forest).

Additional Mann–Whitney tests allowed us to detect differences between sites for individual species’ densities, with values adjusted per trap. We found significant differences for the following species: *Xylosandrus compactus* (*U* = 0, c.v. = 8; Figure 7a), *H. birmanus* (Eichhoff) (= *H. farinosus* Blandford; *U* = 2, c.v. = 8; Figure 7b), *H. eruditus* Westwood (*U* = 0, c.v. = 8; Figure 7c), and *Xylosandrus crassiusculus* (Motschulsky) (*U* = 0, c.v. = 8; Figure 7d).

**Figure 8:**
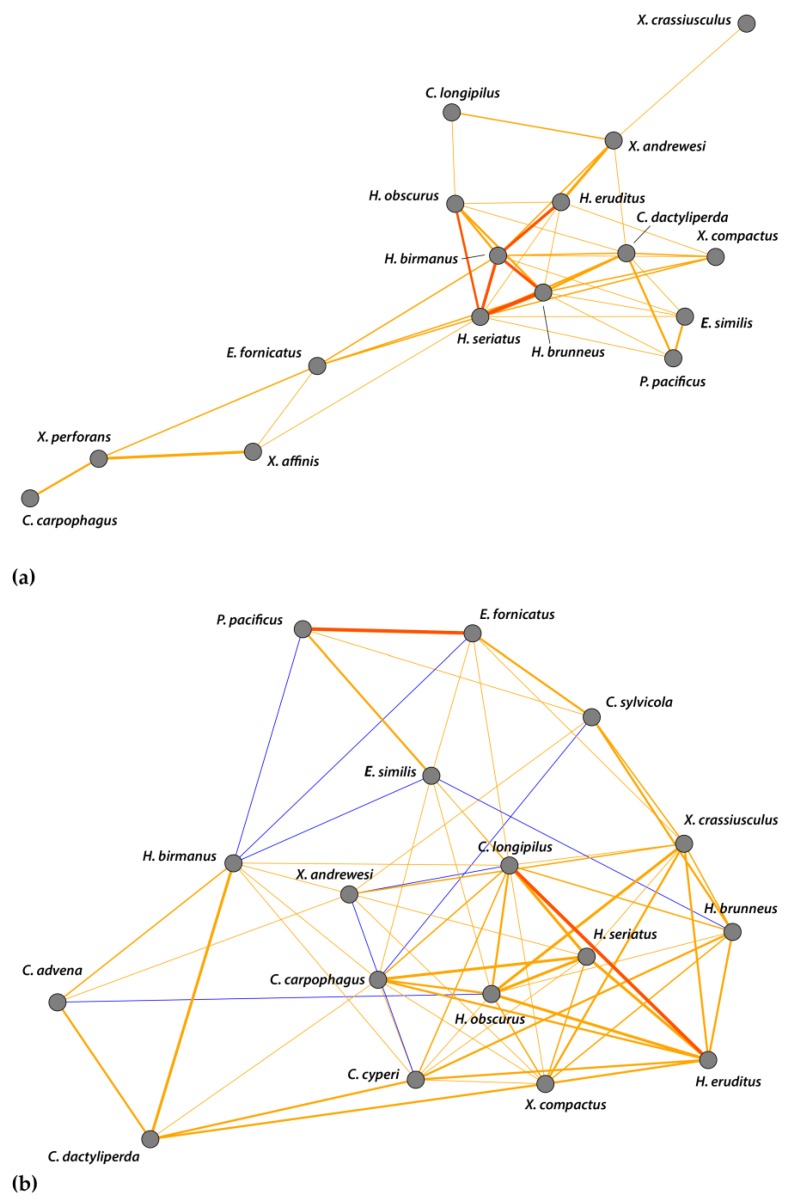
Correlation network of log-transformed abundance data for scolytine species at (**a**) the Kauaʻi Coffee Company and (**b**) Moloaʻa Bay Coffee. Thickness of edges (i.e., lines connecting nodes) varies with strength of correlations; network shows only *R*^2^ > 0.35. Weak trends (0.1 > *p* > 0.05) are yellow (positive) or blue (negative), and significant positive correlations (*p* < 0.05) are orange. There are no significant negative correlations.

To address any influence of beetle–beetle interactions on the communities, we constructed scale-free correlation networks. Figure 8 shows only relationships wherein *R*^2^ > 0.35, and displays significant correlations (*p* < 0.05) alongside weak trends (0.1 > *p* > 0.05) to give a more complete depiction of the community structure. There were no significant negative correlations at either site. At Numila (Figure 8a), *H. birmanus* had significant correlations with *H. brunneus* (*R*^2^ = 0.82), *H. eruditus* (*R*^2^ = 0.74), and *H. seriatus* (Eichhoff) (*R*^2^ = 0.79). *Hypothenemus seriatus* also had significant correlations with *H. brunneus* (*R*^2^ = 0.98) and *H. obscurus* (*R*^2^ = 0.71). At Moloaʻa (Figure 8b), we found significant correlations between *C. longipilus* and *H. eruditus* (*R*^2^ = 0.93), and between *E. fornicatus* and *Ptilopodius pacificus* Schedl (*R*^2^ = 0.97). The strong correlations present at Numila were weak at Moloaʻa, and those in Moloaʻa were not present in Numila.

## 4. Discussion

We found a significant negative correlation between precipitation and total scolytine community abundance (Figure 5). Although this phenomenon initially appears to be a lag of scolytine success following coffee productivity, which itself would lag after a period of heavier rain [1], some data contradict this speculation. Increases in scolytine abundance in Numila in February and August of 2016, for example, did not follow rainy months, and increases that did follow rainy periods tended to be a few months later (Figure 4). Another explanation for this trend is that some scolytines could prefer drier weather. Spatial distribution modeling in Kenya by Jaramillo et al. [12] indicated that *Hypothenemus hampei* is more successful in dry climates than in wet ones.

Our data show that other scolytine species are also more abundant in such conditions; supporting the pattern of the overall community, we also found significant inverse correlations with precipitation for four species: *H. obscurus*, *H. brunneus*, *Cryphalus longipilus*, and *Xyleborinus andrewesi* (Figure 6). Moreover, the scale-free interspecies correlation networks found no significant positive correlations between these four species (Figure 8), which demonstrates that they each have independent relationships with rainfall and that other disparate factors influence their numbers. This indicates that the correlations shown in the interspecies networks provide additive effects to the overall community structure, alongside the importance of rainfall, rather than reflect an indirect effect of species responding to the same forces.

In parsing out the independent variables in a manner similar to Park and Reid [30], we surprisingly found no correlation between beetle abundances and either temperature or harvest times, despite harvest techniques being one of the most obvious differences between the sites. This may hint that scolytines in these communities have little to do with coffee fruit, but we occasionally reared *H. obscurus*, *Xylosandrus compactus*, *Xyleborus affinis* Eichhoff, and *Coccotrypes carpophagus* (Hornung) from coffee fruit collected at these estates.

Despite the correlation between overall scolytine density and precipitation, this study could not find a correlation between diversity and precipitation. Nevertheless, diversity and evenness of the community at Numila tended to decline as the total abundance of scolytines increased (Figure 3 and Figure 4). In February 2016 and January 2017, total scolytine abundance peaked when the community was less diverse. This is appears to be owing to the proportional dominance of *H. obscurus* (Figure 1 and Figure 2), although we found no negative correlations between it and other species (Figure 8a). Species evenness was conversely highest in the summer months when overall scolytine abundance was relatively low for the year. During the summer of 2015 and November 2016, dominance was supplanted by *H. eruditus* and *H. birmanus*, respectively (Figure 2), periods which correspond to episodes of high evenness at Numila. While the data from Moloaʻa depict a rise in diversity alongside a rise in overall abundance in January 2017, peak evenness for the community was in October 2016 when the abundance was lowest.

The Bray–Curtis index found the scolytine communities at Moloaʻa and Numila to be markedly dissimilar in terms of species composition, although the unpaired Mann–Whitney *U* test showed that the communities are proportionally similar in terms of overall scolytine density and species richness. This suggests that the difference between the two communities is how individual species within the community respond to ecological factors. Certain species demonstrate higher success at one site over the other. For instance, *H. obscurus* was the third most abundant species in Moloaʻa but was by far the most dominant in Numila, and *H. brunneus* was the fifth most numerous scolytine in Numila but eighth in Moloaʻa (Figure 1). We furthermore discovered the following four species to have significantly higher relative abundance at Moloaʻa than at Numila: *Xylosandrus compactus*, *X. crassiusculus*, *H. birmanus*, and *H. eruditus* (Figure 7). None of the latter four species displayed a relationship with the abiotic variables measured in this study (e.g., precipitation), so their distribution must be influenced by other factors. Their success at Moloaʻa could be attributed to the few non-coffee crops available there; for instance, *X. compactus* and *X. crassiusculus* are able to use cacao as a host [11,31]. Pérez-De La Cruz et al. [32] list cacao as the host for several species, including *H. eruditus* and *Xyleborus affinis*, the latter of which we found in Numila but not Moloaʻa. Moreover, these authors do not list cacao as a host for *H. birmanus*, whose distribution favored Moloaʻa. Thus, the marginal non-coffee crops at Moloaʻa may not entirely explain the relative success of species there. Another contributing factor may be their lack of a relationship to rainfall. Our results indicate that species such as *H. obscurus* would be constrained by wetter environments, in which others could surpass them, whereas in drier environments such species would be unhindered. Nonetheless, in investigating how much of the dynamics are due to scolytine–scolytine interactions, the scale-free correlation networks detected no significant negative correlations (Figure 8), which may suggest that there is no direct competition among scolytine species within these communities, or that their niches within the coffee estates are segregated.

Unlike Reich et al. [13], we encountered no positive correlations between rainfall and any species in this study, although overall patterns of diversity would be complicated by species that have variable trends (e.g., unaffected). In Brazil, for instance, Morales et al. [33] described an increase in population size of *X. affinis* during decreased rainfall, but they found a positive correlation with rainfall for other xyleborines, complicating community trends. When comparing scolytine communities in Europe and North America, Marini et al. [3] found complex patterns in which precipitation has a significant positive influence on species numbers. Moreover, Hulcr et al. [2] found in Thailand that scolytine communities in wetter environments (i.e., high humidity) exhibited significantly higher diversity than those in dry environments, which were instead dominated by few pantropical pests. Gordon et al. [34] likewise determined that unshaded coffee farms in México had lower diversity but higher abundance than shaded coffee farms, suggesting that communities in warm, dry climates could also be dominated by a few species. The general trends in this study suggest a similar process on Kauaʻi, that some dominant species do well in dry climates, where they outcompete other scolytines, and in wetter periods different species can rise in numbers.

This study additionally appears to track the establishment of two species at the Kauaʻi Coffee Company: *H. seriatus* and *H. brunneus*, the latter of which is a new island record for Kauaʻi. Both were first detected in Numila in July 2016, and progressed to become, respectively, the third and fifth most numerous species of the 2016–2017 harvest season (Figure 2). *Hypothenemus brunneus* appeared at Moloaʻa Bay Coffee at the same time in our data, but unfortunately the shorter sampling period there precluded determining whether this cryphaline was present a priori. If *H. brunneus* was extant in Numila during the first year of sampling, it would have been at exceedingly low densities. This biodiversity study also extended the known distributions for several other species [15,24,35,36,37,38], resulting in additional new records for Kauaʻi, detailed alongside vouchers in S1.

## 5. Conclusions

On Kauaʻi we found a unique opportunity to observe two sites that are similar in many dimensions (e.g., crops, soil, temperature, surrounding vegetation, scolytine density, scolytine richness) but differed in key factors (e.g., harvest method, precipitation, marginal crops). Our findings demonstrate that precipitation regime and species composition determine scolytine community dynamics; certain species have an inverse relationship with rainfall (such as those in Figure 6), which will dominate communities in drier environments/periods, but will be displaced by other species (such as those in Figure 7) in wetter environments/periods. Communities with such members will experience an overall increase in density in drier periods/environments (Figure 4 and Figure 5). Marginal crops may be an additional factor in determining the community composition. Knowledge of these relationships could prove useful to management strategies, because pest management can be targeted in response to scolytine numbers, such as when they are at low densities, depending on the control method. These trends can also help predict the establishment of future invasive species; scolytines such as *H. hampei* that have an inverse relationship with wet climates [12] would be more successful in drier regions like Numila and less so in wetter ones like Moloaʻa. Furthermore, this data could be incorporated into climatic modeling to determine the potential future spatiotemporal distributions of scolytines in Hawaiʻi as climate change progresses [12,39]. Scolytines that have low or nonexistent densities in wetter climates may be able to expand into such areas as the climate changes.

## Supplementary Materials

The following are available online at http://www.mdpi.com/2075-4450/9/4/123/s1. S1: Bark beetle vouchers with notes for new Kauaʻi adventive and naturalization records. Table S1: No statistical relationship between log-transformed monthly scolytine abundances and log-transformed monthly averages of temperature in bark beetle communities on Kauaʻi.
