# Supplementary material for: The Structure and Phenology of Non-Native Scolytine Beetle Communities in Coffee Plantations on Kauaʻi
Source: Insects. 2018 Sep 20;9(4):123. doi: 10.3390/insects9040123 (PMC6315950; doi:10.3390/insects9040123)
Supplement: Supplementary file 1 [file insects-09-00123-s001.zip › Supplemental Table S1.docx]

**Table S1:** No statistical relationship between log-transformed monthly scolytine abundances and log-transformed monthly averages of temperature in bark beetle communities on Kauaʻi. These are values for the overall community as well as the most prominent species individually; other species were too rare to analyze.

| Species | *df* | Test statistic | *p*-value |
| --- | --- | --- | --- |
| *Cryphalus longipilus* | 22 | *F* = 2.65 | 0.12 |
| *Hypothenemus birmanus* | 18 | *F* = 2.16 | 0.16 |
| *Hypothenemus brunneus* | 13 | *τ_b_* = 0.17 | 0.40 |
| *Hypothenemus eruditus* | 22 | *τ_b_* = 0.00 | 1.00 |
| *Hypothenemus obscurus* | 22 | *F* = 0.03 | 0.86 |
| *Xyleborus affinis* | 15 | *τ_b_* = -0.02 | 0.92 |
| *Xyleborinus andrewesi* | 22 | *τ_b_* = -0.05 | 0.78 |
| *Xylosandrus compactus* | 22 | *τ_b_* = -0.27 | 0.08 |
| *Xylosandrus crassiusculus* | 22 | *τ_b_* = -0.13 | 0.41 |
| Overall Moloaʻa community | 6 | *τ_b_* = -0.52 | 0.10 |
| Overall Numila community | 15 | *τ_b_* = -0.07 | 0.72 |
| Total scolytine community | 22 | *F* = 1.85 | 0.19 |
