# Supplementary material for: The Structure and Phenology of Non-Native Scolytine Beetle Communities in Coffee Plantations on Kauaʻi
Source: Insects. 2018 Sep 20;9(4):123. doi: 10.3390/insects9040123 (PMC6315950; doi:10.3390/insects9040123)
Supplement: Supplementary file 1 [file insects-09-00123-s001.zip › Supplemental S1.docx]

**Supplemental 1: bark beetle vouchers with notes for new Kauaʻi adventive and naturalization records**

*Collection acronyms:* University of Hawaiʻi Insect Museum in Honolulu (UHIM), Bernice Pauahi Bishop Museum in Honolulu (BPBM).

*Kauaʻi record:* a species already recorded on other Hawaiian Islands but not previously known on Kauaʻi.

*Adventive:* a non-native species that is new to an area but not yet known to have established populations capable to reproducing.

*Naturalized:* a non-native species which evidently forms established populations.

***Coccotrypes advena* Blandford** **New Kauaʻi Adventive Record**[= *Dendrurgus philippinensis* Eggers; *Thamnurgides tutuilensis* Beeson; *Coccotrypes philippinensis* Schedl; *Poecilips niger* Schedl (full synonymy in Wood [24])]

This species is distinguished from other congeners in the Hawaiian Islands by the lack of asperites on the pronotum [24]. It is established on Oʻahu and Hawaiʻi Island, but is previously unknown to Kauaʻi [15]. We found only 1 specimen, which is insufficient to claim it as established on Kauaʻi.

*Vouchered specimen:* **Hawaiian Islands:** Kauaʻi: Moloaʻa (UTM: 4Q 466194 E, 2453713 N), in Brocap trap in coffee plantation, coll. J. Bernard, 19-XII-2016, det. C. Ewing (1 ♀ in 95% EtOH, UHIM).

***Coccotrypes carpophagus* (Hornung)**[= *Coccotrypes pygmaeus* Eichhoff; *C. punctulatus* Eggers; *C. ceylonicus* Schedl; *C. exasperatus* Schedl (full synonymy in Wood [24])]

Known in the Hawaiian Islands on Hawaiʻi Island, Oʻahu, and Kauaʻi [15].

*Vouchered specimen:* **Hawaiian Islands:** Kauaʻi: Moloaʻa (UTM: 4Q 466139 E, 2453836 N), in Brocap trap in coffee plantation, coll. J. Bernard, 17-I-2017, det. J. Bernard, confirmed by C. Ewing (1 ♀ in 95% EtOH, UHIM).

***Coccotrypes cyperi* (Beeson)** **New Kauaʻi Adventive Record**[= *Thamnurgides indicus* Eggers; *Coccotrypes insularis* Eggers; *Poecilips caraibicus* Schedl; *P. pilifrons* Browne (full synonymy in Wood [24])]

*C. cyperi* can be identified by coarse asperites covering the broadly convex pronotum, which bears a smooth anterior margin, and by the lack of strial setae [24]. This species has never been collected on Kauaʻi before, and is previously known only on Hawaiʻi Island [15]. Because we collected only 1 specimen, we are listing it as adventive rather than naturalized.

*Vouchered specimen:* **Hawaiian Islands:** Kauaʻi: Moloaʻa (UTM: 4Q 466194 E, 2453713 N), in Brocap trap in coffee plantation, coll. J. Bernard, 31-I-2017, det. C. Ewing (1 ♀ pinned, UHIM).

***Coccotrypes dactyliperda* (Fabricius)**[= *Coccotrypes moreirai* Eggers; *C. tanganus* Eggers; *C. elaeocarpi* Beeson (full synonymy in Wood [24])]

Known in the Hawaiian Islands on Hawaiʻi Island, Maui, Oʻahu, and Kauaʻi [15].

*Vouchered specimen:* **Hawaiian Islands:** Kauaʻi: Moloaʻa (UTM: 4Q 466139 E, 2453836 N), in Brocap trap in coffee plantation, coll. J. Bernard, 19-XII-2016, det. J. Bernard, confirmed by C. Ewing (1 ♀ in 95% EtOH, UHIM).

***Cryphalus longipilus* Schedl**  **New Kauaʻi Naturalization Record**[= *Ericryphalus longipilus* (Schedl)]

This species is distinguished from the congeners in the Hawaiian Islands by having no clavate or scale-like setae on the pronotum [23]. It has been collected across the Hawaiian Islands, but distribution remains unreported. We demonstrate here that it is common in plantations on Kauaʻi.

*Vouchered specimens:* **Hawaiian Islands:** Kauaʻi: Numila (UTM: 4Q 447858 E, 2421109 N), in Brocap trap in coffee plantation, coll. J. Bernard, 31-I-2017, det. J. Bernard, corrected from *Ericryphalus* by C. Ewing, confirmed by A. Johnson (1 ♀ in 95% EtOH, UHIM); Numila (UTM: 4Q 442085 E, 2421780 N), in Brocap trap in coffee plantation, coll. J. Bernard, 31-I-2017, det. J. Bernard, corrected from *Ericryphalus* by C. Ewing, confirmed by A. Johnson (1 ♀ in 95% EtOH, UHIM).

***Cryphalus sylvicola* (Perkins)**[= *Ericryphalus sylvicola* Perkins]

Known in the Hawaiian Islands on Hawaiʻi Island, Maui, Lānaʻi, Oʻahu, and Kauaʻi [15].

*Vouchered specimens:* **Hawaiian Islands:** Kauaʻi: Moloaʻa (UTM: 4Q 466194 E, 2453713 N), in Brocap trap in coffee plantation, coll. J. Bernard, 7-XI-2016, det. J. Bernard, confirmed by C. Ewing (3 ♀ in 95% EtOH, UHIM).

***Euwallacea fornicatus* (Eichhoff)**[= *Xyleborus fornicatus* Eichhoff; *X. fornicatior* Eggers; *X. perbrevis* Schedl (full synonymy in Wood [24])]

Known in the Hawaiian Islands on Hawaiʻi Island, Maui, Molokaʻi, Oʻahu, and Kauaʻi [15].

*Vouchered specimen:* **Hawaiian Islands:** Kauaʻi: Moloaʻa (UTM: 4Q 466139 E, 2453836 N), in Brocap trap in coffee plantation, coll. J. Bernard, 1-XI-2016, det. J. Bernard, confirmed by C. Ewing (1 ♀ in 95% EtOH, UHIM).

***Euwallacea similis* (Ferrari)**  **New Kauaʻi Naturalization Record**[= *X. similis* Ferrari [38], *X.* *dilatatulus* Schedl, *X. novaguineanus* Schedl, *X. capito* Schaufuss (additional synonymy in Wood [24])]

This species can be separated from other congeners in Hawaiʻi by the presence of a prominent tubercle in the first interstriae of the elytral declivity [14]. Known in the Hawaiian Islands on Hawaiʻi Island, Maui, Oʻahu, Kauaʻi, and Niʻihau [15].

*Vouchered specimens:* **Hawaiian Islands:** Kauaʻi: Numila (UTM: 4Q 440175 E, 2422266 N), in Brocap trap in coffee plantation, coll. J. Bernard, 26-VII-2016, det. C. Ewing (1 ♀ in 95% EtOH, UHIM); Moloaʻa (UTM: 4Q 466194 E, 2453713 N), in Brocap trap in coffee plantation, coll. J. Bernard, 22-VIII-2016, det. J. Bernard, confirmed by C. Ewing (1 ♀ in 95% EtOH, UHIM); Numila (UTM: 4Q 442088 E, 2421861 N), in Brocap trap in coffee plantation, coll. J. Bernard, 29-IX-2016, det. J. Bernard, confirmed by C. Ewing (1 ♀ in 95% EtOH, UHIM); Numila (UTM: 4Q 447858 E, 2421109 N), in Brocap trap in coffee plantation, coll. J. Bernard, 11-X-2016, det. C. Ewing (1 ♀ in 95% EtOH, UHIM).

***Hypothenemus birmanus* (Eichhoff)**[= *Triarmocerus birmanus* Eichhoff; *Stephanoderes alter* Eggers; *S. castaneus* Wood (see extended synonymy in Wood [24]); *H. farinosus* Blandford]

Known on all the main Hawaiian Islands except Kahoʻolawe [15].

*Vouchered specimens:* **Hawaiian Islands:** Kauaʻi: Numila (UTM: 4Q 440175 E, 2422266 N), in Brocap trap in coffee plantation, coll. J. Bernard, 5-VII-2016, det. J. Bernard, confirmed by C. Ewing (1 ♀ in 95% EtOH, UHIM); Numila (UTM: 4Q 442029 E, 2421883 N), in Brocap trap in coffee plantation, coll. J. Bernard, 26-VII-2016, det. J. Bernard, corrected from *H. farinosus* by C. Ewing (1 ♀ in 95% EtOH, UHIM); Numila (UTM: 4Q 442029 E, 2421883 N), in Brocap trap in coffee plantation, coll. J. Bernard, 6-XII-2016, det. J. Bernard, corrected from *H. farinosus* by C. Ewing (1 ♀ in 95% EtOH, UHIM); Numila (UTM: 4Q 442085 E, 2421780 N), in Brocap trap in coffee plantation, coll. J. Bernard, 18-I-2017, det. J. Bernard, confirmed by C. Ewing (1 ♀ in 95% EtOH, UHIM).

***Hypothenemus brunneus* (Hopkins)**  **New Kauaʻi Naturalization Record**[= *Stephanoderes brunneus* Hopkins, *S. frontalis* Hopkins, *H. cryphalomorphus* Schedl (full synonymy in Wood [24])]

This species was discovered on Molokaʻi in 2016 as a new state record for the Hawaiian Islands [37], which may coincide with its appearance on Kauaʻi; we first detected it concurrently in Moloaʻa and Numila in July 2016. *H. brunneus* likely also occurs on other islands between Molokaʻi and Kauaʻi. The only other congener in Hawaiʻi that bears a transverse carina on the frons is *H. areccae* (Hornung), from which *H. brunneus* is distinguished by having 2–4 large teeth on the anterior margin of its pronotum, having interstriae ⪆ 2× width of striae, and having sparse short ground vestiture on its elytral declivity [24]. It is evidently well-established across Kauaʻi as it was common at both sites in this study.

*Vouchered specimens:* **Hawaiian Islands:** Kauaʻi: Numila (UTM: 4Q 442085 E, 2421780 N), in Brocap trap in coffee plantation, coll. J. Bernard, 6-XII-2016, det. C. Ewing & J. Bernard with guidance from K. Arakaki (1 ♀ in 95% EtOH, UHIM); Numila (UTM: 4Q 442072 E, 2421841 N), in Brocap trap in coffee plantation, coll. J. Bernard, 20-XII-2016, det. J. Bernard with guidance from K. Arakaki (1 ♀ pinned, BPBM); Numila (UTM: 4Q 442085 E, 2421780 N), in Brocap trap in coffee plantation, coll. J. Bernard, 31-I-2017, det. C. Ewing & J. Bernard with guidance from K. Arakaki (1 ♀ in 95% EtOH, UHIM); Moloaʻa (UTM: 4Q 466194 E, 2453713 N), in Brocap trap in coffee plantation, coll. J. Bernard, 21-II-2017, det. J. Bernard with guidance from K. Arakaki (1 ♀ pinned, BPBM).

***Hypothenemus eruditus* Westwood**[= *Cryphalus aspericollis* Wollaston; *H. insularis* Perkins; *H. longipilus* Schedl (full synonymy in Wood [24])]

Known in the Hawaiian Islands on Hawaiʻi Island, Maui, Oʻahu, Kauaʻi, Laysan Atoll, and Kure Atoll [15].

*Vouchered specimen:* **Hawaiian Islands:** Kauaʻi: Numila (UTM: 4Q 447858 E, 2421109 N), in Brocap trap in coffee plantation, coll. J. Bernard, 6-XII-2016, det. J. Bernard, confirmed by C. Ewing (1 ♀ in 95% EtOH, UHIM).

***Hypothenemus obscurus* (Fabricius)**[= *Hylesinus obscurus* Fabricius; *Stephanoderes asperulus* Eichhoff; *Hypothenemus emarginatus* Schedl (full synonymy in Wood [24])]

Known on all the main Hawaiian Islands [36].

*Vouchered specimens:* **Hawaiian Islands:** Kauaʻi: Numila (UTM: 4Q 442029 E, 2421883 N), in Brocap trap in coffee plantation, coll. J. Bernard, 1-XI-2016, det. J. Bernard, confirmed by C. Ewing (1 ♀ in 95% EtOH, UHIM); Numila (UTM: 4Q 442030 E, 2421833 N), in Brocap trap in coffee plantation, coll. J. Bernard, 31-I-2017, det. J. Bernard, confirmed by C. Ewing (1 ♀ in 95% EtOH, UHIM).

***Hypothenemus seriatus* (Eichhoff)**[= *Stephanoderes seriatus* Eichhoff; *S. minutus* Hopkins; *S. hawaiiensis* Schedl (full synonymy in Wood [24])]

Known in the Hawaiian Islands on Hawaiʻi Island, Oʻahu, and Kauaʻi [15].

*Vouchered specimens:* **Hawaiian Islands:** Kauaʻi: Numila (UTM: 4Q 442050 E, 2421771 N), in Brocap trap in coffee plantation, coll. J. Bernard, 6-XII-2016, det. J. Bernard, confirmed by C. Ewing (1 ♀ in 95% EtOH, UHIM); Numila (UTM: 4Q 442029 E, 2421883 N), in Brocap trap in coffee plantation, coll. J. Bernard, 20-XII-2016, det. J. Bernard, confirmed by C. Ewing (1 ♀ in 95% EtOH, UHIM).

***Ptilopodius pacificus* Schedl**

Known in the Hawaiian Islands on Lānaʻi, Oʻahu, and Kauaʻi [15].

*Vouchered specimens:* **Hawaiian Islands:** Kauaʻi: Numila (UTM: 4Q 442293 E, 2421546 N), in Brocap trap in coffee plantation, coll. J. Bernard, 6-XII-2016, det. J. Bernard (1 ♀ in 95% EtOH, UHIM); Moloaʻa (UTM: 4Q 466194 E, 2453713 N), in Brocap trap in coffee plantation, coll. J. Bernard, 29-XI-2016, det. J. Bernard (1 ♀ in 95% EtOH, UHIM).

***Xyleborinus andrewesi* (Blandford)**  **New Kauaʻi Naturalization Record**

This species can be distinguished from the other congener in the Hawaiian Islands, *X. saxesenii* (Ratzeburg) by the acutely tapered elytra. It is previously known to occur on Hawaiʻi Island and Oʻahu [37], but has yet to be recorded on Kauaʻi. Our community assessment shows *X. andrewesi* to be common in plantations on Kauaʻi.

*Vouchered specimens:* **Hawaiian Islands:** Kauaʻi: Moloaʻa (UTM: 4Q 466139 E, 2453836 N), in Brocap trap in coffee plantation, coll. J. Bernard, 7-XI-2016, det. C. Ewing (1 ♀ in 95% EtOH, UHIM); Moloaʻa (UTM: 4Q 466194 E, 2453713 N), in Brocap trap in coffee plantation, coll. J. Bernard, 6-XII-2016, det. C. Ewing (1 ♀ in 95% EtOH, UHIM).

***Xyleborus affinis* Eichhoff**[= *X. sacchari* Hopkins; *X. societatis* Beeson; *X. proximus* Eggers (full synonymy in Wood [24])]

Known in the Hawaiian Islands on Hawaiʻi Island, Maui, Oʻahu, and Kauaʻi [15].

*Vouchered specimens:* **Hawaiian Islands:** Kauaʻi: Numila (UTM: 4Q 442072 E, 2421841 N), in Brocap trap in coffee plantation, coll. J. Bernard, 26-VII-2016, det. C. Ewing (1 ♀ in 95% EtOH, UHIM); Numila (UTM: 4Q 447858 E, 2421109 N), in Brocap trap in coffee plantation, coll. J. Bernard, 11-X-2016, det. C. Ewing (1 ♀ in 95% EtOH, UHIM).

***Xyleborus perforans* (Wollaston)**[= *X. mascarensis* Eichhoff]

Known in the Hawaiian Islands on Hawaiʻi Island, Maui, Oʻahu, Kauaʻi, and Niʻihau [15].

*Vouchered specimens:* **Hawaiian Islands:** Kauaʻi: Numila (UTM: 4Q 442293 E, 2421546 N), in Brocap trap in coffee plantation, coll. J. Bernard, 27-VI-2016, det. J. Bernard (1 ♀ in 95% EtOH, UHIM); Numila (UTM: 4Q 444677 E, 2424562 N), in Brocap trap in coffee plantation, coll. J. Bernard, 12-VII-2016, det. C. Ewing (1 ♀ in 95% EtOH, UHIM).

***Xylosandrus compactus* (Eichhoff)**[= *Xyleborus compactus* Eichhoff; *Xyleborus morstatti* Hagedorn]

Known on all the main Hawaiian Islands except Kahoʻolawe and Niʻihau [15].

*Vouchered specimens:* **Hawaiian Islands:** Kauaʻi: Numila (UTM: 4Q 447858 E, 2421109 N), in Brocap trap in coffee plantation, coll. J. Bernard, 20-XII-2016, det. J. Bernard, confirmed by C. Ewing (1 ♀ in 95% EtOH, UHIM); Numila (UTM: 4Q 440175 E, 2422266 N), in Brocap trap in coffee plantation, coll. J. Bernard, 18-I-2017, det. J. Bernard, confirmed by C. Ewing (1 ♀ in 95% EtOH, UHIM).

***Xylosandrus crassiusculus* (Motschulsky)**[= *Phlaeotrogus crassiusculus* Motschulsky; *Xyleborus semigranosus* Blandford; *Xyleborus crassiusculus* Beaver (see extended synonymy in Samuelson [14])]

Known in the Hawaiian Islands on Hawaiʻi Island, Maui, Oʻahu, and Kauaʻi [15].

*Vouchered specimens:* **Hawaiian Islands:** Kauaʻi: Moloaʻa (UTM: 4Q 466194 E, 2453713 N), in Brocap trap in coffee plantation, coll. J. Bernard, 19-XII-2016, det. J. Bernard, confirmed by C. Ewing (1 ♀ in 95% EtOH, UHIM); Moloaʻa (UTM: 4Q 466194 E, 2453713 N), in Brocap trap in coffee plantation, coll. J. Bernard, 17-I-2017, det. J. Bernard, confirmed by C. Ewing (1 ♀ in 95% EtOH, UHIM).

***Xylosandrus morigerus* (Blandford)** **New Kauaʻi Adventive Record**[= *Xyleborus luzonicus* Eggers, *Xyleborus coffeae* Wurth]

This species differs from other congeners in the Hawaiian archipelago by having a convex elytral base and steep truncated elytral declivity [14]. This is the first record of it occurring on Kauaʻi. Because we detected only a single specimen, found at the Kauaʻi Coffee Company, we cannot yet consider it established. However, it is established on Hawaiʻi Island and Oʻahu [37].

*Vouchered specimens:* **Hawaiian Islands:** Kauaʻi: Numila (UTM: 4Q 442189 E, 2421430 N), in Brocap trap in coffee plantation, coll. J. Bernard, 20-XII-2016, det. J. Bernard, confirmed by C. Ewing (1 ♀ in 95% EtOH, UHIM).
